# Low Serum High-Density Lipoprotein Cholesterol Levels Associate with the *C9orf72* Repeat Expansion in Frontotemporal Lobar Degeneration Patients

**DOI:** 10.3233/JAD-190132

**Published:** 2019-10-29

**Authors:** Olli Jääskeläinen, Eino Solje, Anette Hall, Kasper Katisko, Ville Korhonen, Mika Tiainen, Antti J. Kangas, Seppo Helisalmi, Maria Pikkarainen, Anne Koivisto, Päivi Hartikainen, Mikko Hiltunen, Mika Ala-Korpela, Hilkka Soininen, Pasi Soininen, Annakaisa Haapasalo, Anne M. Remes, Sanna-Kaisa Herukka

**Affiliations:** a Institute of Clinical Medicine–Neurology, University of Eastern Finland, Kuopio, Finland; b Neuro Center, Kuopio University Hospital, Kuopio, Finland; c Institute of Clinical Medicine – Neurosurgery, University of Eastern Finland, Kuopio, Finland; d NMR Metabolomics Laboratory, School of Pharmacy, University of Eastern Finland, Kuopio, Finland; e Computational Medicine, Faculty of Medicine, University of Oulu and Biocenter Oulu, Oulu, Finland; f Institute of Biomedicine, University of Eastern Finland, Kuopio, Finland; g Medical Research Council Integrative Epidemiology Unit at the University of Bristol, Bristol, UK; h Population Health Science, Bristol Medical School, University of Bristol, Bristol, UK; i Systems Epidemiology, Baker Heart and Diabetes Institute, Melbourne, VIC, Australia; j Department of Epidemiology and Preventive Medicine, School of Public Health and Preventive Medicine, Faculty of Medicine, Nursing and Health Sciences, The Alfred Hospital, Monash University, Melbourne, VIC, Australia; k A.I. Virtanen Institute for Molecular Sciences, University of Eastern Finland, Kuopio, Finland; l Medical Research Center, Oulu University Hospital, Oulu, Finland; m Research Unit of Clinical Neuroscience, Neurology, University of Oulu, Oulu, Finland

**Keywords:** *C9orf72* protein, cholesterol, frontotemporal dementia, frontotemporal lobar degeneration, inflammation, lipoproteins

## Abstract

Decreased levels of serum high-density lipoprotein (HDL) cholesterol have previously been linked to systemic inflammation and neurodegenerative diseases, such as Alzheimer’s disease. Here, we aimed to analyze the lipoprotein profile and inflammatory indicators, the high-sensitivity C-reactive peptide (hs-CRP) and glycoprotein acetyls (GlycA), in sporadic and *C9orf72* repeat expansion-associated frontotemporal lobar degeneration (FTLD) patients. The *C9orf72* hexanucleotide repeat expansion is the most frequent genetic etiology underlying FTLD. The concentrations of different lipid measures in the sera of 67 FTLD patients (15 *C9orf72* repeat expansion carriers), including GlycA, were analyzed by nuclear magnetic resonance spectroscopy. To verify the state of systemic inflammation, hs-CRP was also quantified from patient sera. We found that the total serum HDL concentration was decreased in *C9orf72* repeat expansion carriers when compared to non-carriers. Moreover, decreased concentrations of HDL particles of different sizes and subclass were consistently observed. No differences were detected in the very low- and low-density lipoprotein subclasses between the *C9orf72* repeat expansion carriers and non-carriers. Furthermore, hs-CRP and GlycA levels did not differ between the *C9orf72* repeat expansion carriers and non-carriers. In conclusion, the HDL-related changes were linked with *C9orf72* repeat expansion associated FTLD but were not seen to associate with systemic inflammation. The underlying reason for the HDL changes remains unclear.

## INTRODUCTION

Frontotemporal lobar degeneration (FTLD) is the second most common cause of dementing diseases in working-age people and accounts for approximately 10% of all progressive dementias [1]. FTLD is clinically divided into two main subcategories, namely behavioral variant frontotemporal dementia (bvFTD) [2] and primary progressive aphasias (PPAs) [3]. PPAs are further divided into the following subcategories: nonfluent variant primary progressive aphasia (nfvPPA) and semantic variant primary progressive aphasia (svPPA). In addition, the logopenic variant of primary progressive aphasia (lvPPA) is clinically considered a subtype of PPA, but is neuropathologically associated with Alzheimer’s disease (AD) [3].

FTLD presents autosomal dominant inheritance in up to 50% of patients [4, 5]. The most common genetic etiology underlying FTLD is the hexanucleotide repeat expansion (GGGGCC) on the short arm of chromosome 9 open reading frame 72 (*C9orf72*) [6, 7]. This repeat expansion accounts for approximately 25% of familial FTLD cases in Europe and the USA [8]. Besides FTLD, the *C9orf72* repeat expansion also causes up to 40% of familial amyotrophic lateral sclerosis (ALS) cases in these populations [8]. Investigations in induced pluripotent stem cell-derived neurons from *C9orf72* repeat expansion carriers and different animal models have suggested that both toxic gain-of-function and loss-of-function mechanisms underlie *C9orf72* repeat expansion-associated FTLD and ALS [9]. Transcription and aberrant repeat-associated non-ATG (RAN) translation of the expanded *C9orf72* hexanucleotide repeat in both sense and antisense directions have been shown to lead to the formation and accumulation of expanded repeat-containing RNA foci and dipeptide-repeat proteins (DPRs) and result in neurotoxicity and neurodegeneration. In addition, several studies have shown that *C9orf72* repeat expansion carriers display an approximately 50% decrease in the levels of normal *C9orf72* RNA and protein, indicating haploinsufficiency as another potential contributor to disease pathogenesis [9].

Dysfunction in brain lipid homeostasis is suggested to be a risk factor for different neurodegenerative disorders [10, 11]. Altered blood lipid metabolism is known to associate with cardiovascular diseases, well-known risk factors for neurodegenerative diseases, but also with neurodegenerative diseases themselves, even though it is presently unclear if the blood and brain lipid levels correlate with each other. Lowered serum high-density lipoprotein (HDL) cholesterol has been indicated to be linked to AD [12, 13]. In addition, a decreased HDL concentration is related to systemic inflammation [14]. Recent studies *C9orf72* knockout mice have shown drastic systemic inflammation and autoimmune disease-like phenotypes. These examinations together with human studies suggest a potential role for inflammation in *C9orf72* repeat expansion-associated disease pathogenesis [15–17]. So far only a few studies have provided insight into the lipid metabolism in FTLD patients and these studies have not contained analyses of the genetic background of the patients [18, 19]. However, the examination of lipid and cholesterol changes in ALS, a close pathological analogue to *C9orf72* repeat expansion-associated FTLD, has been more extensive [20–30]. Dyslipidemia in ALS has also been acknowledged [21, 29, 30].

Here, our aim was to examine potential alterations in the serum lipoprotein levels in FTLD patients carrying or not the *C9orf72* repeat expansion. To our knowledge, these are the first reported findings that compare lipoprotein alterations in *C9orf72* repeat expansion carriers to non-carriers.

## MATERIALS AND METHODS

### Ethical considerations

The study was performed according to the principles of the Declaration of Helsinki. Written informed consent was obtained from the participants. The study protocol was approved by the Research Ethics Committee of the Northern Savo Hospital District.

### Patients

A cohort comprising a total of 67 patients with FTLD, diagnosed between the years 1996–2017 at the memory outpatient clinics of Kuopio University Hospital, was utilized in this study (Table 1). An experienced neurologist, specialized in cognitive and dementing disorders, examined all of the patients. The patients with bvFTD were diagnosed according to the latest diagnostic criteria by Rascovsky and colleagues [2], and patients with PPAs were diagnosed according to the Gorno-Tempini diagnostic criteria [3]. A retrospective review based on these same criteria was used for patients who had been originally diagnosed before the Rascovsky or Gorno-Tempini criteria were published. All patients with bvFTD, nfvPPA, or svPPA fulfilled the criteria with either a probable or a definite diagnosis. Patients with FTLD-MND had at least a probable diagnosis of bvFTD, nfvPPA, or svPPA and the clinical manifestation of motoneuron disease (MND). None of the patients in our cohort were diagnosed with lvPPA. The serum samples were collected at the time of diagnosis. Collected samples were aliquoted and frozen (–80°C) for future analyses.

**Table 1:** Demographic data and prevalence of the *C9orf72* repeat expansion and *APOE* *ɛ4* alleles among diagnosed FTLD patients

	bvFTD	PPA (nfv + sv)	FTLD-MND	Total
Cases	49	12	6	67
Sex, f/m	30/19 (61/39%)	7/5 (58/32%)	4/2 (67/33%)	41/26 (61.2/38.8%)
Mean age at diagnosis, y	65.0	69.8	61.8	65.6
*C9orf72* carriers/non-carriers	14/21 (40/60% ^*^)	1/10 (1/10% ^*^)	0/5 (0/100% ^*^)	15/36 (29/61% ^*^)
*APOE* *ɛ*4 carriers/non-carriers	15/22 (40.5/ 59.5% ^*^)	2/8 (20/80% ^*^)	0/3 (0/100% ^*^)	17/33 (34/66% ^*^)
	*C9orf72* repeat	*C9orf72* repeat
	expansion carriers	expansion non-carriers
*APOE* *ɛ*4 carriers/non-carriers	3/9 (25/75% ^*^)	9/14 (39.1/60.9% ^*^)

^*^Ratio of genotyped cases. *APOE* *ɛ4*, Apolipoprotein E *ɛ*4; bvFTD, behavioral variant frontotemporal dementia; *C9orf72*, Chromosome 9 open reading frame 72; FTDL-MND, Frontotemporal lobar degeneration – motoneuron disease; PPA (nfv + sv), Primary progressive aphasia (non-fluent variant + semantic variant).

Patients with FTLD were further divided into two subgroups based on their *C9orf72* repeat expansion status: *C9orf72* repeat expansion carriers (N = 15) and *C9orf72* repeat expansion non-carriers (N = 36). Within the clinical subgroups, 14 bvFTD and one nfvPPA patient carried the *C9orf72* repeat expansion. None of the FTLD-MND or svPPA patients carried the *C9orf72* repeat expansion. Genotyping data of the *C9orf72* repeat expansion was not available for 23.9% (16/67) FTLD patients (13 bvFTD, one nfvPPA, and one FTLD-MND). Demographic information of the study patients is presented in Table 1.

### Genetic analyses

The *C9orf72* repeat expansion was analyzed using the repeat-primed polymerase chain reaction assay [6]. One patient with the *C9orf72* expansion carried an intermediate expansion (28 repeats) and the rest had the full expansion (>40 repeats). The patients classified as *C9orf72* repeat expansion non-carriers had less than five repeats. Other genes associated with FTLD were not analyzed as mutations in these genes have previously been observed to be extremely rare in the Finnish population [31, 32].

The apolipoprotein E (*APOE*) alleles were genotyped from extracted genomic DNA with polymerase chain reaction using TaqMan genotyping assays (rs429358 and rs7412 polymorphisms, Applied Biosystems, Foster City, CA, USA) and allelic discrimination (ABI 7500 platform) [33]. The QIAamp DNA blood mini extraction kit (QIAGEN) was used to extract genomic DNA from blood.

### Lipoprotein analysis

The concentrations for lipoprotein particles, phospholipids, free cholesterol, cholesterol esters, and triglycerides in the sera for each lipoprotein size and density were quantified using a nuclear magnetic resonance spectroscopy platform [34, 35]. The value for total lipids in each lipoprotein subclass is calculated by summing phospholipids, total cholesterol, and triglycerides. The concentration for total cholesterol is the sum of free cholesterol and cholesterol esters. This platform has been used in multiple genetic and epidemiological setups [36–39]. Lipoprotein data was missing from one *C9orf72* repeat expansion carrier and from one non-carrier (both diagnosed with bvFTD). This same method was used to quantify glycoprotein acetyls (mainly a1-acid glycoprotein) (GlycA) from the patient sera. Circulating GlycA are a prospective and novel biomarker for systemic inflammation [40].

### High sensitivity C-reactive protein

To analyze the state of inflammation in the study cohort, high sensitivity C-reactive protein (hs-CRP) was quantified from patient serum with Cobas 8000 (Hitachi High Technology Co, Tokyo, Japan), using a high sensitive assay for CRP (Latex CRPHS, cat# 04628918 190, Cobas c systems, Roche Diagnostics GmbH, Mannheim, Germany).

### Statistics

An independent two sample *t*-test was used to test the significance of differences between the *C9orf72* repeat expansion carriers and non-carriers. Hs-CRP median levels were compared with independent samples Mann-Whitney U-test.

The normality of distributions was tested by calculating the skewness of data and comparing it to the skewness value after logarithmic transformation. Abnormal distribution of a variable was corrected using logarithmic transformation if the absolute difference in skewness between logarithmic and native data was greater than one. The threshold for statistical significance was *p* < 0.05.

**Fig.1:**
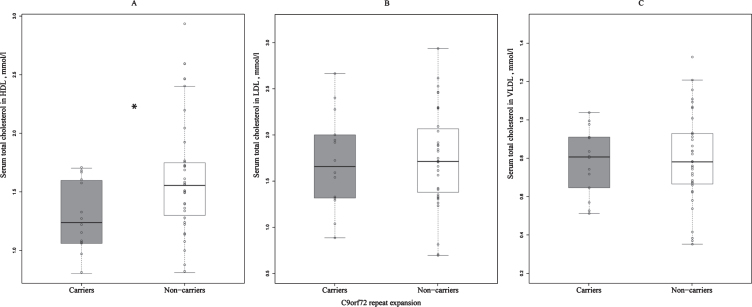
Differences in the concentrations of serum total cholesterol in HDL (A), LDL (B), and VLDL (C) between *C9orf72* repeat expansion carriers and non-carriers. Total serum cholesterol concentrations in HDL are significantly lower in the repeat expansion carriers when compared to non-carriers. ^*^statistically significant difference, *p* = 0.030; *C9orf72*, Chromosome 9 open reading frame 72; HDL, high-density lipoprotein; LDL, low-density lipoprotein; VLDL, very low-density lipoprotein.

Statistical analysis was conducted using SPSS (v23 and v25, IBM Analytics, Armonk, NY, USA). Figure 1 was generated with R/Rstudio (v3.4.1, Boston, MA, USA) and ggplot2 third-party R package (v2.2.1).

## RESULTS

We found that the concentration of total HDL cholesterol in the sera of *C9orf72* repeat expansion carriers was lower when compared to non-carriers (*p* = 0.030) (Fig. 1A). Interestingly, there were no differences in the serum concentrations of total LDL and VLDL cholesterol between the *C9orf72* carriers and non-carriers (Fig. 1B, C).

Similar findings were observed when the subclasses of HDL, LDL, and VLDLs of different particle sizes were analyzed. The concentration of total cholesterols in very large HDLs was lower in the *C9orf72* repeat expansion carriers when compared to non-carriers (*p* = 0.004). Moreover, the concentrations of phospholipids (*p* = 0.010) and free cholesterol (*p* = 0.007) as well as total lipids (*p* = 0.047) in very large HDLs were also lower. Particle concentration, and concentrations of cholesterol esters and triglycerides in very large HDLs did not differ between the *C9orf72* repeat expansion carriers and non-carriers. In addition, differences in the concentrations of subclasses in large HDLs were similar to those in the very large HDL subclasses. The *C9orf72* repeat expansion carriers showed lower concentrations of total lipids (*p* = 0.046), phospholipids (*p* = 0.041), free cholesterol (*p* = 0.046), cholesterol esters (*p* = 0.031), and large HDL particles (*p* = 0.033) compared to non-carriers. However, the concentrations of total cholesterol and triglycerides in large HDLs were not altered between the *C9orf72* repeat expansion carriers and non-carriers. In the medium and small HDL subclasses, only the concentration of free cholesterol in small HDLs was lower in the *C9orf72* repeat expansion carriers compared to non-carriers (*p* = 0.037) (Table 2). Concentrations of LDL subclasses were similar between the *C9orf72* repeat expansion carriers and non-carriers. Likewise, there were no differences in the VLDL subclasses between the *C9orf72* repeat expansion carriers and non-carriers. The ratio of free cholesterol and cholesterol esters in very large HDLs were lower in the *C9orf72* repeat expansion carriers (*p* = 0.010). There were no other clear differences in this ratio in any other HDL subclass or in any VLDL or LDL subclasses.

**Table 2:** Differences in particle concentration, total lipids, phospholipids, total cholesterol, cholesterol esters, free cholesterol, and triglycerides in very large, large, medium, and small high-density lipoproteins between carriers and non-carriers of the *C9orf72* repeat expansion

HDL particle size	HDL subclass	Mean. *C9orf72* carriers, mmol/l	Mean. *C9orf72* non-carriers, mmol/l	*p*
Very large	Particle concentration	3.56e-04	5.28e-04	0.055
	Total lipids	0.36	0.54	***0***.***047***
	Phospholipids	0.14	0.23	***0***.***010***
	Total cholesterol	0.20	0.29	***0***.***004***
	Cholesterol esters	0.16	0.21	0.073
	Free cholesterol	0.049	0.081	***0***.***007***
	Triglycerides	0.020	0.023	0.406
Large	Particle concentration	1.01e-03	1.42e-03	***0***.***033***
	Total lipids	0.64	0.89	***0***.***046***
	Phospholipids	0.30	0.43	***0***.***041***
	Total cholesterol	0.30	0.43	0.052
	Cholesterol esters	0.23	0.32	***0***.***031***
	Free cholesterol	0.068	0.10	***0***.***046***
	Triglycerides	0.034	0.041	0.357
Medium	Particle concentration	1.81e-03	2.08E-03	0.112
	Total lipids	0.77	0.89	0.106
	Phospholipids	0.37	0.42	0.108
	Total cholesterol	0.37	0.44	0.094
	Cholesterol esters	0.29	0.34	0.100
	Free cholesterol	0.077	0.095	0.091
	Triglycerides	0.036	0.033	0.359
Small	Particle concentration	4.69e-03	4.90e-03	0.374
	Total lipids	1.04	1.09	0.333
	Phospholipids	0.57	0.60	0.376
	Total cholesterol	0.43	0.45	0.374
	Cholesterol esters	0.32	0.33	0.731
	Free cholesterol	0.10	0.12	***0***.***037***
	Triglycerides	0.047	0.046	0.735
	Total cholesterol in HDL	1.3	1.6	***0***.***030***
	HDL particle diameter	9.9 nm	10.1 nm	***0***.***035***

*C9orf72*, Chromosome 9 open reading frame 72; HDL, high-density lipoprotein. The *p*-values are presented as native values and are not subjected to multiple test.

The mean diameter of HDL particles was smaller in patients carrying the *C9orf72* repeat expansion in comparison to non-carriers (*p* = 0.035). The mean diameters of VLDL and LDL particles were similar between the *C9orf72* repeat expansion carriers and non-carriers. Moreover, the concentrations of phosphatidylcholine and conjugated linoleic acid were lower in cases carrying the *C9orf72* repeat expansion compared to non-carriers (*p* = 0.023 and *p* = 0.040, respectively). We observed that the concentration of triglycerides in small and medium HDL particles was higher in carriers of two *APOE* *ɛ4* alleles when compared to patients with only one or no *APOE* *ɛ4* alleles. The *APOE* *ɛ4* genotype did not affect the concentrations of HDLs in any other subclass. In addition, we saw that serum HDL concentrations were consistently lower in men in nearly every subclass. Furthermore, serum triglycerides in large and small LDL were also lower in men.

The median hs-CRP concentration in the *C9orf72* repeat expansion carriers was 0.60 pg/ml. There was no difference between the median hs-CRP concentrations between *C9orf72* repeat expansion carriers and non-carriers (median hs-CRP in non-carriers was 0.65 pg/ml). The non-carriers showed slightly higher mean value, but this was due to one bvFTD patient with hs-CRP concentration of 36.40 pg/ml. In addition, there were three non-carriers whose hs-CRP concentrations were under the quantification range of the assay (0.15 pg/ml). These values were set to zero. The hs-CRP levels did not correlate with HDL or LDL concentrations in any lipoprotein subclass in carriers and non-carriers combined, but were observed to negatively correlate with extremely large, very large, and large VLDLs in multiple subclasses. We also observed a non-significant trend of slightly depressed GlycA in FTLD patients with the *C9orf72* repeat expansion when compared to those without (*p* = 0.064). The mean concentration of GlycA in the *C9orf72* repeat expansion carriers was 1.49 mmol/l and 1.62 mmol/l in non-carriers.

## DISCUSSION

We found here that *C9orf72* repeat expansion carriers show lower concentrations of HDL category lipids as compared to non-carriers. This alteration in serum lipids appears to be specific for HDL as no changes were observed in the concentrations of VLDL or LDL between these groups. To our knowledge, this is the first study reporting blood lipoprotein alterations in *C9orf72* repeat expansion associated FTLD patients. Dyslipidemia has been suggested to associate with brain atrophy and is thought to occur in neurodegenerative diseases, i.e., AD, but not much information is available on lipid alterations in FTLD. Growing data suggest that a low concentration of serum HDL may be associated with an increased risk of certain neurodegenerative diseases. For instance, a lowered HDL serum cholesterol has been described to associate with AD [12, 13]. However, contradictory evidence has also been presented, linking an increased risk of AD and cholesterol esters relative to total lipids in large HDL [41].

Our results are in line with previous findings related to lipoprotein changes in FTLD [18, 19, 42]. It has been previously reported that bvFTD patients have a lower blood HDL cholesterol concentration when compared to AD patients [18]. The same study also reported an increased total cholesterol/HDL ratio in bvFTD patients as compared to a healthy control group. In addition blood triglycerides were elevated in both bvFTD and SD patients compared to a control group [18]. In a more recent study, the same group reported increased triglycerides and a total cholesterol/HDL ratio, and a lowered HDL cholesterol concentration in ALS and FTD patients when compared to controls [43]. Furthermore, a recent lipidomics analysis from plasma showed alterations in different lipid species in bvFTD as compared to AD patients or controls and suggested hypertriglyceridemia and hypoalphalipoproteinemia in bvFTD [19]. Several studies also associate the incidence and risk of ALS to changes in LDL category cholesterols and lipids [20, 21, 23, 27, 29]. The information regarding the involvement of HDL in ALS is inconclusive. None of these studies, however, have contained genetic analyses to allow comparisons between *C9orf72* repeat expansion carriers and non-carriers. In the study by Wuolikainen et al., the *C9orf72* repeat expansions were confirmed for eleven ALS patients, but the cholesterol variables were not examined on the basis of the repeat expansion carrier status [22].

Chronic inflammation has also been reported to decrease serum HDL levels [14]. However, the mechanism by which inflammation may alter lipid metabolism is not fully understood. As HDL has been shown to possess anti-inflammatory and antioxidant properties and is a central transporter of cholesterol from the tissues to the liver, inflammation might compromise this reverse transporter function and thus promote cholesterol accumulation and exacerbate inflammatory responses, creating a vicious cycle (see, e.g., the review by Feingold and Grunfeld in 2016 [44]). Recently, a plausible association of inflammatory disturbances and FTLD has been proposed. First, an increased prevalence of autoimmune diseases has been reported in FTLD patients compared to controls [45–47] and second, FTLD seems to be inversely linked to cancer [48], which may suggest alterations in the immune system or responses. Interestingly, the contribution of autoimmune mechanisms and genetic variation in loci associated with the immune system in FTLD were also suggested in recent studies [15, 49–51]. In line with these patient-derived data, *C9orf72* repeat expansion has been linked to disturbances in the immune system [52], e.g., in mouse models with loss of function of the *C9orf72* gene, which indicate a severe autoimmune phenotype, high mortality rate, and increased levels of proinflammatory cytokines and signs of neuroinflammation [16, 17, 52, 53]. On the other hand, a remarkable amount of FTLD patients display dietary changes [2], which may partly be related to changes in serum lipid concentrations. The *C9orf72* repeat expansion associated FTLD patients have been reported to exhibit less alterations in eating habits than repeat expansion non-carriers. In particular the acquired preference for sweet foods was lower in *C9orf72* carriers [54]. As we did not detect alterations in lipid particles other than HDL, we suggest that eating habits do not underlie the decreased serum HDL levels in our study.

Connections between HDLs and inflammation are not possible to evaluate with our findings. Based on the hs-CRP and GlycA levels there were no differences in relation to the *C9orf72* repeat expansion in the FTLD patients. This could, at least partly be due to the low sample size, but could also reflect the mechanistic complexity underlying the HDL-related changes and chronic inflammation. It is also well known that certain common autoimmune disorders, e.g., rheumatoid arthritis, may not necessarily increase CRP levels. Furthermore, there are no previous reports regarding hs-CRP or GlycA in FTLD or their correlations with the *C9orf72* repeat expansion. Lunette and colleagues connected ALS progression with hs-CRP [55], but also inconclusive results have been presented [56]. In the latter study, Nagel et al. did not see an association between hs-CRP levels and the risk of ALS. Currently, GlycA has been associated with systemic inflammation [40, 57] but has not been as extensively studied in the context of neurodegeneration. A study by Tynkkynen et al. assessed inflammation in AD and incident dementia by quantifying GlycA but reported only weak associations [41]. Further research is warranted to better elucidate these apparently complex associations.

Of the tested cofounding factors, sex significantly affected the concentrations of lipoprotein variables in the sera. Men showed lower concentrations of multiple HDL category lipids when compared to women. However, the prevalence of the *APOE* *ɛ4* allele was only affecting these HDL levels in two subclasses. Considering the similarity in sex distributions among *C9orf72* repeat expansion carriers and non-carriers, we do not suspect that the reported changes in HDL category lipids between *C9orf72* repeat expansion carriers and non-carriers are due to sex-related differences in HDL or lipoprotein metabolism.

### Strengths and weaknesses

The strengths of this study include utilization of a clinically and genetically well-characterized FTLD patient cohort with a large number of *C9orf72* repeat expansion carriers. However, even though the proportion of *C9orf72* expansion carriers in the cohort is high, the size of the total cohort is limited. Thus, larger sample sets are needed to confirm the results obtained in the study. Lipoprotein quantification provides absolute concentrations, instead of relying on relative concentrations or signal intensities. The serum metabolomics platform is thoroughly validated and used extensively. Another limiting factor is the fact that the provided *p*-values were not subjected to multiple testing. After applying multiple correction, the *p*-values depicted non-significant findings, due to the small sample size. However, a clearly discernable trend is obvious and warrants further research to thoroughly validate the presented association.

### Conclusions

We conclude that total serum HDL cholesterol was lower in FTLD patients with the *C9orf72* repeat expansion. This was consistent in several subclasses of very large and large HDLs. There were no significant differences in the concentrations of VLDL or LDL cholesterol subclasses between *C9orf72* repeat expansion carriers and non-carriers. We did not find an association between these lipoprotein changes and quantifiable markers of systemic inflammation, hs-CRP or GlycA. Thus, the background behind the presented HDL changes remains unclear and needs further investigations.

## Supplementary Material

Supplementary Material
