# Supplementary material for: Low Serum High-Density Lipoprotein Cholesterol Levels Associate with the C9orf72 Repeat Expansion in Frontotemporal Lobar Degeneration Patients
Source: J Alzheimers Dis. 2019 Oct 29;72(1):127–37. doi: 10.3233/JAD-190132 (PMC6839456; doi:10.3233/JAD-190132)
Supplement: Supplementary Material [file jad-72-jad190132-s001.docx]

**Supplementary Material**

**Low Serum High-Density Lipoprotein Cholesterol Levels Associate with the C9orf72 Repeat Expansion in Frontotemporal Lobar Degeneration Patients**

*Mean lipoprotein values in bvFTD, PPA, and FTLD-MND*

File description: Mean concentration of lipoprotein subclasses between behavioral variant frontotemporal lobar dementia (bvFTD), primary progressive aphasia (PPA) and frontotemporal degeneration patients with motoneuron symptoms (FTD-MND). Lipoprotein densities: VLDL, Very low-density lipoproteins; LDL, Low density lipoproteins; HDL, High density lipoproteins. Size groups: XXL, Extremely large; XL, Very large; L, Large; M, Medium; S, Small; XS, Very small. Functional subgroup measures: P, Particle concentration; L, Total lipids; PL, Phospholipids; C, Total cholesterol; CE, Cholesterol esters; FC, Free cholesterol; TG, Triglycerides. The unit for the values is mmol/l (nm for particle diameters). Lipoprotein data was missing from 3 bvFTD patients. Asterisk (*) denotes a significant difference in mean lipoprotein subclass concentration between groups (p < 0.05, one-way ANOVA).

|  | bvFTD | PPA | FTD-MND |
| --- | --- | --- | --- |
| XXL-VLDL-P | 1.32e-07 | 1.18e-07 | 2.18e-07 |
| XXL-VLDL-L | 0.028 | 0.025 | 0.046 |
| XXL-VLDL-PL * | 0.0034 | 0.0032 | 0.0067 |
| XXL-VLDL-C * | 0.0050 | 0.0040 | 0.0087 |
| XXL-VLDL-CE * | 0.0027 | 0.0021 | 0.0043 |
| XXL-VLDL-FC * | 0.0023 | 0.0020 | 0.0044 |
| XXL-VLDL-TG * | 0.021 | 0.018 | 0.040 |
| XL-VLDL-P | 6.67e-07 | 6.65e-07 | 1.24e-06 |
| XL-VLDL-L | 0.065 | 0.065 | 0.12 |
| XL-VLDL-PL * | 0.010 | 0.011 | 0.022 |
| XL-VLDL-C * | 0.014 | 0.012 | 0.028 |
| XL-VLDL-CE * | 0.0072 | 0.0054 | 0.014 |
| XL-VLDL-FC * | 0.0069 | 0.0064 | 0.014 |
| XL-VLDL-TG * | 0.042 | 0.042 | 0.094 |
| L-VLDL-P | 4.98e-06 | 4.90e-06 | 8.95e-06 |
| L-VLDL-L * | 0.29 | 0.28 | 0.51 |
| L-VLDL-PL | 0.049 | 0.048 | 0.088 |
| L-VLDL-C | 0.060 | 0.054 | 0.10 |
| L-VLDL-CE | 0.031 | 0.027 | 0.051 |
| L-VLDL-FC | 0.029 | 0.027 | 0.051 |
| L-VLDL-TG | 0.18 | 0.18 | 0.32 |
| M-VLDL-P | 1.91e-05 | 1.79e-05 | 2.97e-05 |
| M-VLDL-L | 0.64 | 0.59 | 0.98 |
| M-VLDL-PL | 0.12 | 0.11 | 0.18 |
| M-VLDL-C | 0.18 | 0.15 | 0.24 |
| M-VLDL-CE | 0.098 | 0.081 | 0.12 |
| M-VLDL-FC | 0.079 | 0.070 | 0.12 |
| M-VLDL-TG | 0.34 | 0.33 | 0.55 |
| S-VLDL-P | 3.59e-05 | 3.16e-05 | 4.13e-05 |
| S-VLDL-L | 0.72 | 0.63 | 0.80 |
| S-VLDL-PL | 0.16 | 0.14 | 0.17 |
| S-VLDL-C | 0.29 | 0.25 | 0.28 |
| S-VLDL-CE | 0.18 | 0.16 | 0.16 |
| S-VLDL-FC | 0.11 | 0.10 | 0.12 |
| S-VLDL-TG | 0.27 | 0.24 | 0.36 |
| XS-VLDL-P | 4.79e-05 | 4.13e-05 | 4.35e-05 |
| XS-VLDL-L | 0.61 | 0.53 | 0.54 |
| XS-VLDL-PL | 0.18 | 0.16 | 0.15 |
| XS-VLDL-C | 0.30 | 0.26 | 0.26 |
| XS-VLDL-CE | 0.19 | 0.16 | 0.18 |
| XS-VLDL-FC | 0.11 | 0.094 | 0.081 |
| XS-VLDL-TG | 0.13 | 0.11 | 0.14 |
| L-LDL-P | 2.00e-04 | 1.80e-04 | 1.63e-04 |
| L-LDL-L | 1.4 | 1.3 | 1.2 |
| L-LDL-PL | 0.34 | 0.33 | 0.29 |
| L-LDL-C | 0.97 | 0.88 | 0.79 |
| L-LDL-CE | 0.67 | 0.58 | 0.54 |
| L-LDL-FC | 0.30 | 0.30 | 0.24 |
| L-LDL-TG | 0.13 | 0.10 | 0.091 |
| M-LDL-P | 1.61e-04 | 1.47e-04 | 1.32e-04 |
| M-LDL-L | 0.82 | 0.76 | 0.68 |
| M-LDL-PL | 0.21 | 0.21 | 0.19 |
| M-LDL-C | 0.56 | 0.50 | 0.46 |
| M-LDL-CE | 0.39 | 0.33 | 0.31 |
| M-LDL-FC | 0.17 | 0.17 | 0.15 |
| M-LDL-TG | 0.060 | 0.049 | 0.037 |
| S-LDL-P | 1.84e-04 | 1.72e-04 | 1.57e-04 |
| S-LDL-L | 0.52 | 0.49 | 0.45 |
| S-LDL-PL | 0.14 | 0.14 | 0.13 |
| S-LDL-C | 0.34 | 0.32 | 0.28 |
| S-LDL-CE | 0.24 | 0.21 | 0.19 |
| S-LDL-FC | 0.10 | 0.11 | 0.093 |
| S-LDL-TG | 0.039 | 0.034 | 0.031 |
| XL-HDL-P | 4.96e-04 | 4.47e-04 | 4.00e-04 |
| XL-HDL-L | 0.51 | 0.46 | 0.41 |
| XL-HDL-PL | 0.21 | 0.19 | 0.16 |
| XL-HDL-C | 0.28 | 0.26 | 0.23 |
| XL-HDL-CE | 0.20 | 0.18 | 0.17 |
| XL-HDL-FC | 0.072 | 0.076 | 0.061 |
| XL-HDL-TG | 0.024 | 0.018 | 0.022 |
| L-HDL-P | 1.29e-03 | 1.31e-03 | 9.40e-04 |
| L-HDL-L | 0.82 | 0.83 | 0.59 |
| L-HDL-PL | 0.39 | 0.40 | 0.28 |
| L-HDL-C | 0.39 | 0.40 | 0.28 |
| L-HDL-CE | 0.29 | 0.30 | 0.22 |
| L-HDL-FC | 0.092 | 0.097 | 0.062 |
| L-HDL-TG | 0.042 | 0.031 | 0.032 |
| M-HDL-P | 1.91e-03 | 2.18e-03 | 1.78e-03 |
| M-HDL-L | 0.81 | 0.94 | 0.76 |
| M-HDL-PL | 0.39 | 0.43 | 0.36 |
| M-HDL-C | 0.39 | 0.48 | 0.36 |
| M-HDL-CE | 0.30 | 0.38 | 0.29 |
| M-HDL-FC | 0.084 | 0.10 | 0.076 |
| M-HDL-TG | 0.037 | 0.027 | 0.039 |
| S-HDL-P | 4.69e-03 | 5.19e-03 | 4.55e-03 |
| S-HDL-L | 1.04 | 1.16 | 1.01 |
| S-HDL-PL | 0.56 | 0.63 | 0.57 |
| S-HDL-C | 0.43 | 0.48 | 0.39 |
| S-HDL-CE | 0.32 | 0.36 | 0.28 |
| S-HDL-FC | 0.11 | 0.12 | 0.11 |
| S-HDL-TG | 0.05 | 0.04 | 0.05 |
| VLDL particle diameter | 36.6 | 36.6 | 37.9 |
| LDL particle diameter | 23.6 | 23.5 | 23.6 |
| HDL particle diameter | 10.0 | 10.0 | 9.9 |
| Total cholesterol in VLDL | 0.84 | 0.73 | 0.91 |
| Total cholesterol in LDL | 1.9 | 1.7 | 1.5 |
| Total cholesterol in HDL | 1.5 | 1.6 | 1.3 |
